# Supplementary material for: Crosstalk Pathway between Trehalose Metabolism and Cytokinin Degradation for the Determination of the Number of Berries per Bunch in Grapes
Source: Cells. 2020 Oct 29;9(11):2378. doi: 10.3390/cells9112378 (PMC7693805; doi:10.3390/cells9112378)
Supplement: Supplementary file 1 [file cells-09-02378-s001.pdf]

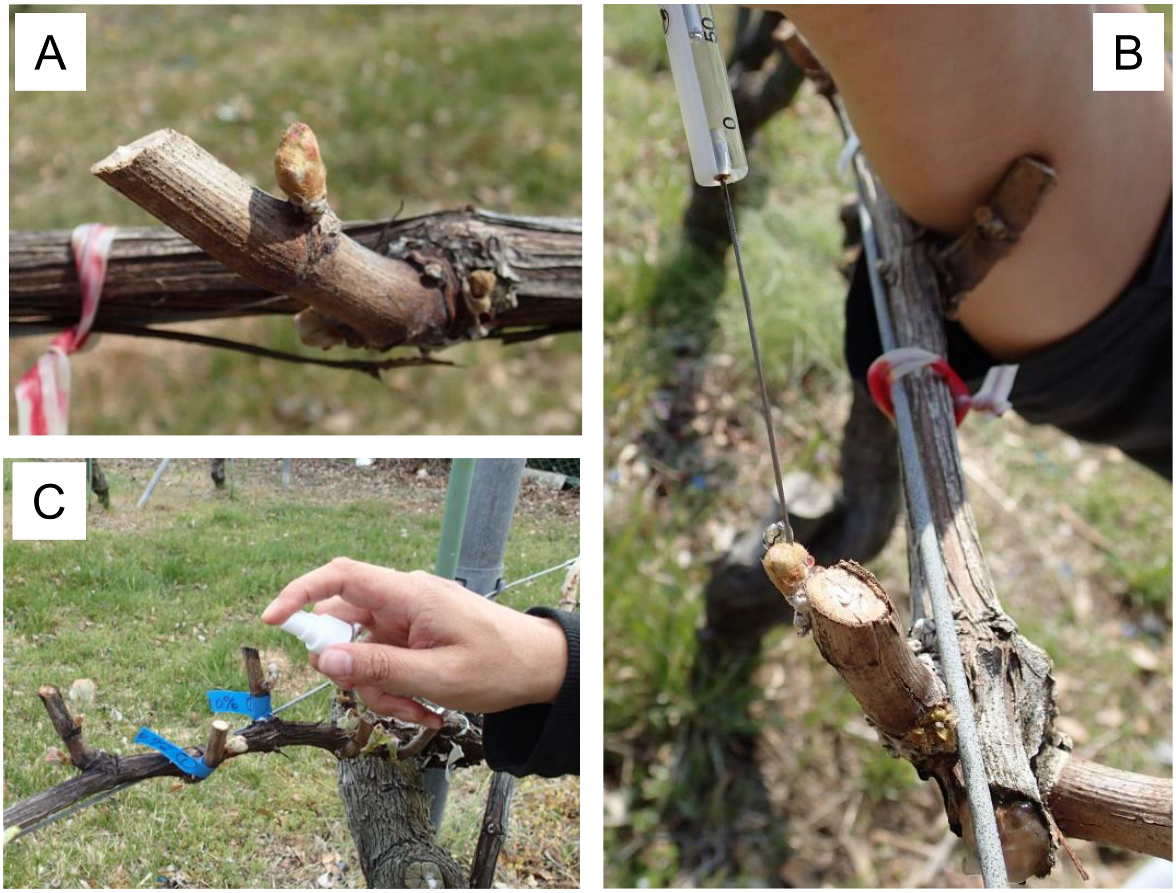

Figure S1. Injection of trehalose into buds of field-grown grapevines using a microsyringe. (A) Buds, nearing bud break (Eichorn-Lorenz Stage 3, brownish wool clearly visible), were obtained from *V. vinifera* cv. Pinot noir grapevines. (B) The buds were treated with trehalose using a microsyringe. (C) After 10 days post treatment, young shoots (Eichorn-Lorenz Stage 5 to7, bud burst or first leaf unfolded) from the buds were again sprayed with trehalose using an atomizer.
